# Chondroblastic Osteosarcoma of the Palate: A Rare Case Report with Diagnostic Considerations 

**DOI:** 10.30476/dentjods.2025.104234.2511

**Published:** 2026-03-01

**Authors:** Iman Mohammadi, Forooz Keshani, Bahareh Fattahi, Mohammad Mehdi Soltani, Arman Roghani

**Affiliations:** 1 Dept. of Oral and Maxillofacial Surgery, Dental Implants Research Center, Dental Research Institute, School of Dentistry, Isfahan University of Medical Sciences, Isfahan, Iran.; 2 Dental Research Center, Dept. of Oral and Maxillofacial Pathology, Dental Research Institute, School of Dentistry, Isfahan University of Medical Sciences, Isfahan, Iran.; 3 Postgraduate Student, Dept. of Oral and Maxillofacial Pathology, School of Dentistry, Isfahan University of Medical Sciences, Isfahan, Iran.; 4 Postgraduate Student, Dept. of Oral and Maxillofacial Surgery, School of Dentistry, Isfahan University of Medical Sciences, Isfahan, Iran.; 5 Postgraduate Student, Dept. of Oral and Maxillofacial Medicine, School of Dentistry, Isfahan University of Medical Sciences, Isfahan, Iran.

**Keywords:** Osteosarcoma, Palatal Osteosarcoma, Chondroblastic osteosarcoma, Hard Palates, Maxilla, Disease Management

## Abstract

Osteosarcoma is a rare and highly malignant primary bone tumor that infrequently occurs in the maxillofacial region. Among these, maxillary osteosarcomas are uncommon, and their occurrence in the palatal region is exceptionally rare, with fewer than five cases reported to date. Chondroblastic osteosarcoma, a subtype characterized by cartilaginous and osteoid components, poses diagnostic challenges due to its histological similarities with other cartilaginous tumors. This study presents a 35-year-old female referred to the Department of Oral and Maxillofacial Surgery at Isfahan University of Medical Sciences with a one-month history of palatal pain and swelling which worsened during eating. Clinical examination revealed a firm, ulcerated mass on the right side of the hard palate, clinically mimicking oral squamous cell carcinoma or salivary gland neoplasms. Radiographic evaluation using cone-beam computed tomography (CBCT) revealed an ill-defined, mixed-density lesion with a moth-eaten appearance, cortical bone destruction, and root resorption of adjacent teeth- features suggestive of an aggressive malignancy. Histopathological examination confirmed the diagnosis of chondroblastic osteosarcoma, a rare histologic subtype characterized by malignant cells producing both osteoid and cartilaginous matrix. Given its resemblance to other cartilaginous tumors, particularly chondrosarcoma, this subtype presents significant diagnostic challenges. The patient underwent hemimaxillectomy, followed by adjuvant chemotherapy. Intraoperative frozen section pathology was used to confirm tumor-free surgical margins- an essential step in reducing recurrence risk. This case underscores the importance of a multidisciplinary approach in the diagnosis and management of rare craniofacial malignancies such as chondroblastic osteosarcoma. Early recognition, accurate diagnosis, and prompt aggressive treatment are crucial for improving patient prognosis and reducing recurrence in such complex and rare presentations.

## Introduction

Osteosarcoma is a rare and highly malignant tumor that originates from bone-forming mesenchymal cells and is considered the most common primary bone malignancy. While it predominantly affects the metaphysis of long bones in adolescents and young adults, gnathic osteosarcomas, especially in the maxilla, are much rarer, accounting for less than 10% of all cases [ 1 - 2 ]. As outlined in Table 1, osteosarcoma can be classified into several clinical subtypes, including conventional osteosarcoma, telangiectatic osteosarcoma, parosteal osteosarcoma, periosteal osteosarcoma, and high-grade surface osteosarcoma [ 2 - 3 ].

**Table 1:** Clinical subtypes of osteosarcoma

Subtype	Histologic Grade	Key Features
Conventional (Osteoblastic, Chondroblastic, Fibroblastic)	High grade	Most common; central tumor with varying matrix production
Telangiectatic	High grade	Blood-filled spaces, mimics cystic lesions; aggressive
Parosteal	Low grade	Surface lesion from outer periosteum; slow-growing
Periosteal	Intermediate grade	Arises from inner periosteal layer; typically involves long bone diaphysis
High-Grade Surface	High grade	Rare, aggressive surface tumor; requires wide resection

Conventional osteosarcoma is the most common type, including osteoblastic, chondroblastic, and fibroblastic variants. Telangiectatic osteosarcoma is characterized by blood-filled spaces and a highly aggressive nature. Parosteal osteosarcoma is a low-grade tumor arising from the outer periosteum. Periosteal osteosarcoma is defined as an intermediate-grade variant affecting the diaphysis of long bones. Finally, high-grade surface osteosarcoma is described as a rare, aggressive tumor requiring extensive surgical resection [ 2 - 3 ].

Although osteosarcomas exhibit considerable histopathologic variation (Table 2), the essential microscopic criterion is direct production of osteoid by malignant mesenchymal cells. In addition to osteoid, the tumor cells may produce chondroid and fibrous connective tissue. Histopathologic findings may range from relatively uniform, round or spindle-shaped cells in low-grade tumors to markedly pleomorphic cells with bizarre nuclear and cytoplasmic shapes in high-grade tumors [ 4 - 5 ].

**Table 2:** Histopathologic subtypes of osteosarcoma

Histopathologic Subtype	Matrix Produced	Cell Morphology	Diagnostic Challenges
Osteoblastic	Osteoid (bone matrix)	Malignant osteoblast-like cells, moderate to severe pleomorphism	Can mimic reactive bone-forming lesions
Chondroblastic	Cartilage and osteoid	Bizarre, pleomorphic cells embedded in chondroid matrix	Resembles chondrosarcoma and benign cartilage tumors [2,3]
Fibroblastic	Fibrous stroma and osteoid	Spindle-shaped fibroblast-like cells with moderate atypia	May resemble fibrosarcoma or desmoplastic lesions
Telangiectatic	Minimal osteoid; blood-filled spaces	Multinucleated giant cells, high mitotic activity	Mimics aneurysmal bone cyst and giant cell tumor
Small Cell	Minimal matrix	Sheets of small round cells	Can be confused with Ewing sarcoma
Low-Grade Central	Osteoid and fibrous matrix	Uniform spindle cells, minimal atypia	Resembles fibrous dysplasia or ossifying fibroma

The chondroblastic subtype of osteosarcoma, characterized by the production of a cartilaginous matrix, is particularly challenging to diagnose due to its histological similarities with benign cartilage tumors. This complicates both the clinical and histological evaluation [ 2 - 3 ]. Osteosarcoma most commonly affects adolescents and young adults, with peak incidence in the second decade of life. In contrast, its occurrence in older adults and in the maxillofacial region is uncommon, accounting for less than 10% of all cases [ 6 ]. Maxillary osteosarcoma, particularly when arising in the palate, is exceptionally rare, with fewer than seven cases reported globally to date. This rarity contributes to significant diagnostic and therapeutic challenges due to its atypical presentation and location [ 6-9 ] (Table 3). This underscores the importance of considering this diagnosis when patients present with palatal masses, even though it is a rare occurrence. The radiologic appearance of osteosarcoma varies depending on the subtype and its location. Typical radiographic features include mixed-density lesions, a sunburst pattern, Codman’s triangle, cortical bone destruction, and a motheaten appearance. Advanced imaging techniques, including magnetic resonance imaging (MRI) and computed tomography (CT) scans, are crucial for assessing the tumor's full extent, including soft tissue involvement and potential metastasis [ 4 ].

**Table 3:** Summary of published case reports of palatal osteosarcoma

Case	Authors	Gender / Age	Tumor Size / Location	Histopathological Subtype	Clinical Outcome	Outcome
1	Yildiz *et al*.[6]	23 -year-old male	Maxilla, posterior and palatal	Fibroblastic Osteosarcoma	Palatal and vestibular swelling. Mucosal ulceration.	No evidence of disease
2	Yildiz *et al*. [6]	22 -year-old male	Palatal large mass (>5 cm) extending to the eye	Osteogenic Osteosarcoma	Destructive lytic irregular lesion	Died of disease 1 year after the operation
3	Yildiz *et al*. [6]	22-year-old male	Maxilla, palatal	Osteosarcoma	Maxillary mass and palatal swelling for two months. History of retinoblastoma when 6 months of age	Lost to follow up
4	Yildiz *et al*. [6]	18-year-old female	Maxilla	Chondroblastic Osteosarcoma	Painful and ulcerated palatal swelling involving tuber maxilla and sinus (>5 cm).	Lost to follow up
5	Hewitt *et al*. 2007 [7]	32 -year-old male	Palatal mass; size not specified	Parosteal Osteosarcoma	Partial maxillectomy; disease-free after 3 years; no adjuvant therapy	No evidence of local recurrence after 3 years
6	Silveria *et al*. [8]	39-year-old female	Maxilla, palatal	Parosteal Osteosarcoma	Large exophytic tumoral mass on the right side of the hard palate no recurrence or alteration	after 4 years
7	Shokri *et al*. [9]	21-year-old male	Maxilla, palatal	Osteosarcoma	Pain and swelling on the left side of palate	Lost to follow up
8	Mohammadi *et al*.	35-year-old female	Maxilla, palatal	Chondroblastic Osteosarcoma	Pain, swelling, and ulceration in the palate	No recurrence and metastasis after 3 months

## Case Presentation

Case Presentation 

A 35-year-old female was referred to the Department of Oral and Maxillofacial Surgery at Isfahan University of Medical Sciences with a chief complaint of pain, swelling, and ulceration in the palate, particularly aggravated during eating. The patient reported that symptoms had begun one month prior and had progressively worsened. The swelling was reported as uncomfortable, though not accompanied by facial asymmetry or paresthesia.

**Figure 1:**
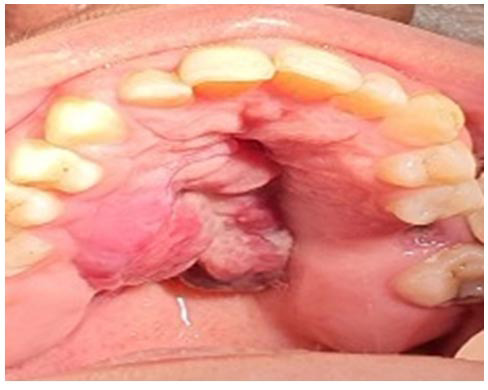
Firm, non-mobile swelling on the right side of the hard palate, with ulcerated surfaces

Clinical examination revealed mucosal erythema and bleeding, though there were no signs of purulent discharge. Her medical history was unremarkable, with no known systemic or hereditary conditions. She was a non-smoker, did not consume alcohol or use recreational drugs, and was only taking folic acid supplements at the time of presentation. On intraoral examination, a firm, non-mobile swelling was detected on the right side of the hard palate, measuring approximately 3×5cm (Figure 1). The lesion was firmly attached to the underlying palatal mucosa, with an ulcerated surface, suggestive of a solid mass. 

At this stage, the differential diagnosis included salivary gland tumors, lymphoma, squamous cell carcinoma, and primary bone tumors [ 3 ]. 

**Figure 2:**
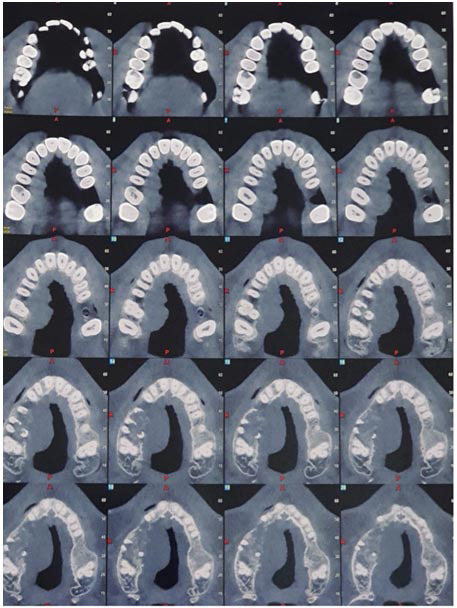
Cone beam computed tomography (CBCT) axial view showing ill-defined mixed-density lesion with a moth-eaten appearance located in the right side of the maxilla

To further evaluate the lesion, a cone-beam computed tomography (CBCT) scan was performed. The axial view revealed an ill-defined lesion in the right maxilla, exhibiting mixed-density characteristics and a classic “moth-eaten” appearance (Figure 2). The panoramic view demonstrated extensive cortical bone destruction in the maxillary region, along with significant root resorption involving the right upper 4th, 5th, and 6th teeth. Additionally, coronal sections showed evidence of palatal soft tissue bulging. In the general view, the CBCT scan showed involvement of the maxilla, palate, and alveolar process to clarify the extent of the tumor. These radiographic findings were strongly indicative of an aggressive malignant process, necessitating further diagnostic work-up [ 10 ]. 

**Figure 3:**
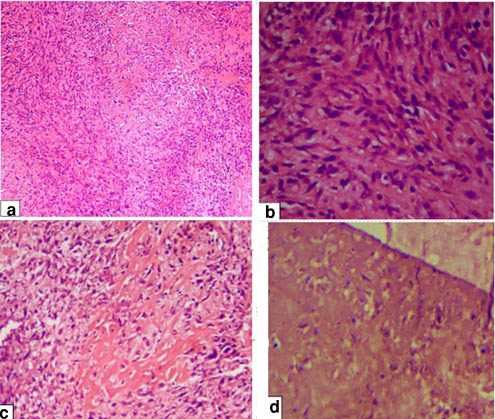
**a:** Micrograph hematoxylin and eosin (H&E) (100×) showing sarcomatous proliferation of malignant spindle cells, **b:** Micrograph H&E (400×) showing cellular and nuclear atypia and pleomorphism with increased nucleus to cell ratio and hyperchromatic nuclei, **c:** Icrograph H&E(200×) showing areas of immature bone(osteoid) formation, **d:** Micrograph H&E(200×) showing areas of immature cartilage(chondroid) formation with larger lacunae

An incisional biopsy was performed to obtain a definitive diagnosis. Histopathological examination using hematoxylin and eosin (H&amp;E) staining revealed a highly cellular and proliferative lesion at 40× magnification. At 100× magnification, malignant spindle-shaped cells exhibiting sarcomatous proliferation and osteoid formation were observed. Islands of immature cartilage (chondroid) formation with prominent lacunae were also noted. The surface epithelium was covered with a fibrino-leukocytic membrane. At higher magnification (400×), mesenchymal cells showed marked cellular and nuclear atypia, pleomorphism, an increased nucleus-to-cytoplasm (N/C) ratio, and hyperchromatic nuclei. These histological features were diagnostic of chondroblastic osteosarcoma (Figure 3). 

Following the confirmation of the diagnosis, the patient underwent an inferior subtotal maxillectomy for complete tumor excision. After standard surgical preparation and draping, general anesthesia was administered. Given the absence of involvement of the maxillary sinus and other superior structures, an intraoral approach was selected for access.

The lesion was initially outlined with a 1 cm safety margin, and the marked borders were sectioned using a surgical saw. The lesion was completely separated from the surrounding structures- including the left pterygoid plate, left zygomaticomaxillary buttress, and right palate up to the dentoalveolar region- using a series of osteotomes. 

**Figure 4:**
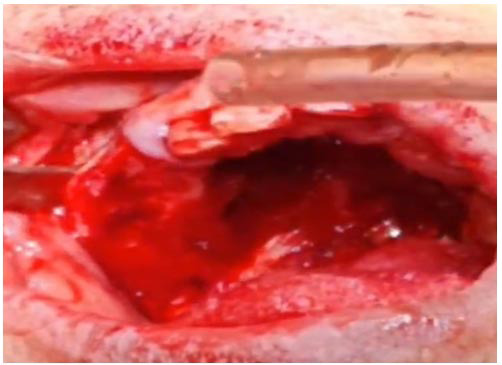
Intraoperative view after lesion resection via safe margin

Considering the aggressive behavior of osteosarcoma and its tendency for local invasion, intraoperative frozen section analysis was utilized to confirm clear surgical margins. This real-time histopathological evaluation plays a critical role in guiding intraoperative decision-making and ensuring oncologic completeness. In this case, the frozen section analysis confirmed that all margins were free of tumor by at least 1 cm. Due to perforation of the nasal floor; a buccal fat pad graft from the right side was used for reconstruction. After achieving hemostasis, the mucosal edges were sutured. A nasogastric tube was placed to facilitate postoperative feeding (Figure 4).

**Figure 5:**
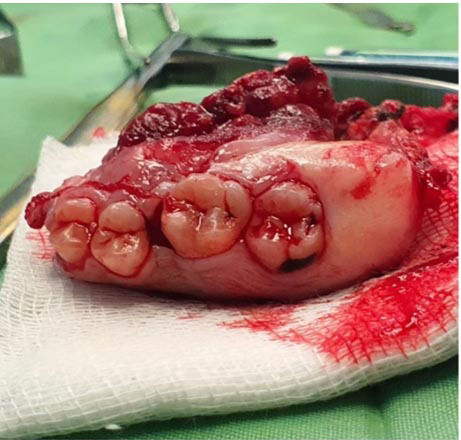
Surgical specimen of the hemi-maxillectomy showing the resected maxillary bone with attached teeth and the tumor mass

The surgical specimen was submitted for further histopathological examination (Figure 5), which revealed a conventional osteosarcoma chondroblastic type. The tumor measured up to 3.5 cm at its greatest dimension, demonstrated high-grade features, and exhibited less than 5% necrosis. No evidence of lymphovascular invasion was found, and all surgical margins were clear, indicating a successful resection. 

Postoperatively, the patient was initiated on a systemic chemotherapy regimen aimed at eliminating potential pulmonary micro-metastases, which are commonly associated with osteosarcoma. The chemotherapy protocol included methotrexate, doxorubicin, and cisplatin, which are standard agents in osteosarcoma treatment [ 10 - 11 ]. 

## Discussion

This case report adds to the limited number of documented instances of chondroblastic osteosarcoma in the maxilla, highlighting its clinical significance and the challenges associated with its diagnosis and management.

The clinical presentation of chondroblastic osteosarcoma in the maxilla can be insidious, with symptoms such as pain, swelling, and ulceration. These nonspecific symptoms often lead to delayed diagnosis, as they can mimic more common conditions. The rapid growth and aggressive nature of the lesion raised suspicion for a malignant process. In this case, the patient presented with a painful, swollen, and ulcerated lesion on the palate, which progressively worsened over a month. At this stage, clinical differential diagnoses included squamous cell carcinoma (SCC), malignant salivary gland tumors, soft tissue tumors, and hard tissue tumors, as the lesion presented as an ulcer with a rolled border.

The lesion’s rapid growth was particularly concerning for a malignant process. Notably, benign salivary gland neoplasms usually do not exhibit such rapid progression, and although rare, the palate is more frequently affected by chondrosarcoma than osteosarcoma [ 4 ].

Radiographic imaging is pivotal in the evaluation of maxillary lesions. In this case, CBCT revealed a mixed-density lesion exhibiting both hypodense and hyper dense areas, with a moth-eaten appearance, marked cortical bone destruction, and extensive root resorption- features indicative of an aggressive malignancy [ 4 ]. These CBCT findings are valuable in differentiating osteosarcoma from less aggressive lesions. However, a definitive diagnosis requires histopathological examination [ 5 ]. The radiological features of craniofacial osteosarcomas closely resemble those of lesions in the appendicular skeleton, typically demonstrating bone destruction with a wide zone of transition, periosteal reaction, and osteoid formation within the tumor matrix [ 5 ]. 

Histopathological analysis in this case confirmed the diagnosis of chondroblastic osteosarcoma, revealing malignant spindle cells, cellular and nuclear atypia, and regions of immature bone (osteoid) and cartilage (chondroid) formation. Immunohistochemical staining is essential for distinguishing chondroblastic osteosarcoma from chondrosarcoma, as chondroblastic osteosarcoma is positive for vimentin, epithelial membrane antigen, S100, and rarely positive for cytokeratin, whereas chondrosarcoma is positive for vimentin and S100 [ 9, 4 ]. The presence of osteoid within the tumor matrix further differentiates chondroblastic osteosarcoma from chondrosarcoma. ​ Furthermore, immunohistochemical staining for IDH1 and IDH2 is crucial for differentiating chondroblastic osteosarcoma from chondrosarcoma, underscoring the importance of accurate tumor classification for guiding treatment decisions and predicting prognosis [ 9, 4 ].

The primary treatment for osteosarcoma, including chondroblastic osteosarcoma, is wide surgical excision to achieve negative margins. In this case, an inferior subtotal maxillectomy (hemimaxillectomy) was performed successfully. Postoperative histopathological findings revealed no lymphovascular invasion and clear surgical margins, which are critical for reducing the risk of local recurrence [ 10 ]. Adjuvant chemotherapy is essential in managing potential micrometastatic disease, commonly involving agents such as methotrexate, doxorubicin, and cisplatin [ 12 ]. This multimodal therapeutic approach, combining surgical resection and chemotherapy, is particularly vital for the aggressive nature of the disease and its tendency for early metastasis, enabling effective disease control and optimal patient management [ 13 ].

The prognosis of maxillofacial osteosarcoma is influenced by several factors, including tumor size and location, histological grade, surgical margins, and response to adjuvant therapy. Early detection and complete surgical resection with clear margins significantly enhance the prognosis [ 14 ]. Nonetheless, due to the high metastatic potential, particularly to the lungs, vigilant long-term follow-up with regular imaging and clinical examinations is imperative. In this case, the patient's postoperative course included chemotherapy aimed at eradicating residual disease and preventing recurrence [ 15 - 16 ].

**Figure 6:**
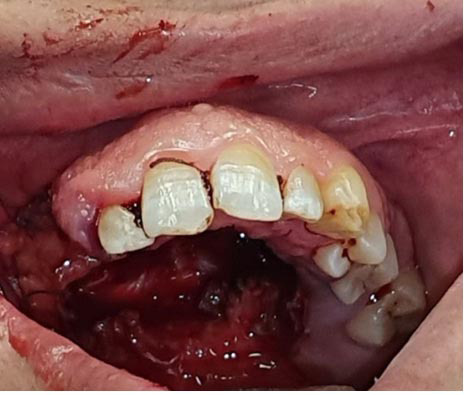
The intraoral photograph showing no evidence of recurrence three months after surgery and following several chemotherapy sessions

The patient was fed via a nasogastric tube for approximately one month after surgery. Afterwards, a soft diet and liquids were continued. Figure 6 was taken about three months after the surgery and following several chemotherapy sessions. A chest X-ray was performed, and there was no evidence of disease recurrence or metastasis. The patient complained of fluid coming out of the nose while eating and has experienced slight weight loss during this period. 

A review of the literature reveals a limited number of reported cases of chondroblastic osteosarcoma in the maxilla. For instance, a study by Rajab *et al*. [ 17 ] reported a 21-year-old female with a 3.3×1.8cm chondroblastic osteosarcoma in the maxilla, treated with chemotherapy followed by surgery, achieving disease-free status at 3 years. In contrast, the present case involved a 35-year-old female with a 5×5cm lesion, treated with chemotherapy, surgery, radiotherapy, and additional chemotherapy, with progression after neoadjuvant chemotherapy. This comparison underscores the variability in clinical presentation, treatment approaches among patients with chondroblastic osteosarcoma in the maxilla. 

Bajpai *et al*.’s study [ 18 ] describes chondroblastic osteosarcoma as “the wolf in sheep’s clothing” because it closely resembles cartilaginous tumors like chondrosarcoma under the microscope. This similarity makes chondroblastic osteosarcoma especially challenging to diagnose correctly [ 18 ]. Similarly, the extensive ulcer and destruction in this case can be shown the aggressive clinical behavior of chondroblastic osteosarcoma. Accurate diagnosis, therefore, depends heavily on histopathological and immunohistochemical assessment to distinguish it from similar lesions, particularly in anatomically complex regions such as the palate.

Although osteosarcoma of the maxilla is rare, its occurrence in the palate is particularly uncommon, with fewer than six cases reported in the literature. To better contextualize our findings, we conducted a review of these reported cases, summarizing patient demographics, tumor size, histopathological subtype, clinical presentation, treatment modality, and outcome. This comparison emphasizes the rarity of our case and highlights the diagnostic and therapeutic considerations specific to palatal involvement (Table 1). This table demonstrates the consistent diagnostic challenge posed by chondroblastic osteosarcoma in this rare location. It also emphasizes the need for heightened clinical suspicion and a multidisciplinary approach for accurate diagnosis and effective management.

According to Table 3, the age of the present case is older than that of the other cases and occurred in the female. Additionally, the presence of ulceration on the lesion could lead to a misdiagnosis by the clinician.

Moreover, it is worth noting that advancements in molecular pathology and targeted therapy are ongoing. Future research could potentially provide new insights into more effective treatment protocols tailored to the individual molecular profiles of tumors, potentially improving outcomes for patients with rare presentations such as palatal osteosarcoma. The written informed consent was obtained from the patient for publication of the details of her medical case and any accompanying images. 

## Conclusion

In conclusion, this case highlights the complexities associated with diagnosing and managing palatal osteosarcoma. Comprehensive radiographic assessment, coupled with histopathological and immunohistochemical analyses, is crucial for accurate diagnosis. A multimodal therapeutic approach, including wide surgical resection and adjuvant chemotherapy, remains the cornerstone of effective management. Ongoing research and long-term follow-up are essential for improving patient outcomes and advancing our understanding of this rare and aggressive malignancy.
